# Analyses of the Large Subunit Histidine-Rich Motif Expose an Alternative Proton Transfer Pathway in [NiFe] Hydrogenases

**DOI:** 10.1371/journal.pone.0034666

**Published:** 2012-04-12

**Authors:** Emma Szőri-Dorogházi, Gergely Maróti, Milán Szőri, Andrea Nyilasi, Gábor Rákhely, Kornél L. Kovács

**Affiliations:** 1 Department of Biotechnology, University of Szeged, Szeged, Hungary; 2 BayGen Institute, Bay Zoltán Foundation for Applied Research, Szeged, Hungary; 3 Department of Chemical Informatics, Juhász Gyula Faculty of Education, University of Szeged, Szeged, Hungary; 4 Institute of Biophysics, Biological Research Centre, Hungarian Academy of Sciences, Szeged, Hungary; Institute of Enzymology of the Hungarian Academy of Science, Hungary

## Abstract

A highly conserved histidine-rich region with unknown function was recognized in the large subunit of [NiFe] hydrogenases. The HxHxxHxxHxH sequence occurs in most membrane-bound hydrogenases, but only two of these histidines are present in the cytoplasmic ones. Site-directed mutagenesis of the His-rich region of the *T. roseopersicina* membrane-attached Hyn hydrogenase disclosed that the enzyme activity was significantly affected only by the replacement of the His104 residue. Computational analysis of the hydrogen bond network in the large subunits indicated that the second histidine of this motif might be a component of a proton transfer pathway including Arg487, Asp103, His104 and Glu436. Substitutions of the conserved amino acids of the presumed transfer route impaired the activity of the Hyn hydrogenase. Western hybridization was applied to demonstrate that the cellular level of the mutant hydrogenases was similar to that of the wild type. Mostly based on theoretical modeling, few proton transfer pathways have already been suggested for [NiFe] hydrogenases. Our results propose an alternative route for proton transfer between the [NiFe] active center and the surface of the protein. A novel feature of this model is that this proton pathway is located on the opposite side of the large subunit relative to the position of the small subunit. This is the first study presenting a systematic analysis of an *in silico* predicted proton translocation pathway in [NiFe] hydrogenases by site-directed mutagenesis.

## Introduction

Hydrogenases are the key enzymes of hydrogen metabolism catalyzing the reversible heterolytic cleavage of molecular hydrogen according to the reaction: H_2_↔2H^+^+2e^−^. These metalloenzymes are widespread in bacteria and archaea and are present in some eukaryotes. Hydrogenases are classified on the basis of the metal content of their active site: [NiFe], [FeFe] or [Fe] hydrogenases [1], [2]. The core of a [NiFe] hydrogenase consists of a small subunit, which is responsible for the electron transfer between the active center and the surface of the enzyme, and a large subunit harboring the binuclear active site [3].

*Thiocapsa roseopersicina* BBS, which belongs to the family of purple sulfur photosynthetic bacteria [4], has been shown to possess four functional [NiFe] hydrogenases with differences in their *in vivo* function, localization and composition [5], [6]. Two of these enzymes (Hyn and Hup) are membrane-associated, while the other two are localized in the cytoplasm (Hox1 and Hox2). Furthermore, the genes of a regulatory hydrogenase (similar to HupUV in *Rhodobacter capsulatus* [7] and HoxBC in *Ralstonia eutropha* [8]) could also be detected in *T. roseopersicina* (*hupTUV*) [7]–[9], but they are not expressed in this organism. The Hyn hydrogenase is a truly bidirectional enzyme with remarkable stability; it is active even when it is extracted from the photosynthetic membrane [10].

The crystal structures of periplasmic [NiFe(Se)] hydrogenases from sulfate-reducing [11]–[15] bacteria and one of the photosynthetic bacteria [16] have been reported. These structures were used to model and study several structure-function relationships of [NiFe] hydrogenases. The structural analysis of the periplasmic [NiFe] hydrogenase of *Desulfovibrio gigas* showed that the metal atoms of the active site are deeply buried inside the protein [11], and that the Ni and Fe are coordinated by cysteine thiolates of the L2 and L5 CxxC motifs [17]. The latter consensus sequence is also involved in the biosynthesis of hydrogenases as the endoproteolytic cleavage of the carboxy-terminus takes place at the Cx_2_Cx_2_H/R motif. The endopeptidases cleave after the conserved His (or Arg) amino acid of this motif and this maturation step is essential for the proper folding and assembly of the large subunit [3]. Following the removal of an approximately 25–32 amino acid fragment from the C-terminus of the protein, the matured large and small subunits form the functional heterodimer.

Furthermore, numerous studies aimed to map the submolecular pathways between the active site and the enzyme surface channeling hydrogen, proton, electron, oxygen and CO [1], [13], [17]–[19].

Molecular dynamics simulations were used to identify possible pathways of molecular hydrogen entering inside the hydrogenase, and to detect the channels potentially involved in transfer of H_2_ to and from the active site. The V67A point mutation in the large subunit of the [NiFe] hydrogenase of *D. gigas* was created and tested *in silico* whether it modulates H_2_ access to the active site. It was suggested that this residue might be a control point in the catalytic mechanism of [NiFe] hydrogenases, and it might also be a controlling element of the access of O_2_ to the active site, where oxygen acts as an inhibitor of the catalytic activity [19].

Molecular H_2_ enters the hydrogenase mainly via hydrophobic channels and the initial site for H_2_ cleavage is the Ni. The resulting electrons and protons are transferred to biological acceptors reaching the surface of the enzyme in distinct atomic pathways [20].

In [NiFe] hydrogenases, electrons are transferred from the active site to the redox partner via a chain of three iron-sulfur (FeS) clusters. A surface-exposed distal [4Fe4S] cluster has an unusual His(Cys)_3_ ligation. The essential function of this residue in determining the rates of inter- and intramolecular electron transfers to and from the distal cluster was demonstrated by site-directed mutagenesis in *D. fructosovorans* [21]. Similar strategy was used to investigate the role of the medial [3Fe4S] cluster in the intramolecular electron transfer. Pro238 of *D. fructosovorans* [NiFe] hydrogenase, which occupies the position of potential ligand of the lacking fourth Fe-site of the [3Fe4S] cluster was replaced by cysteine as in the case of native *Desulfomicrobium baculatum* [NiFeSe] hydrogenase [22]. The results showed no significant alteration of the spectroscopic and redox properties of the two native [4Fe4S] clusters and the [NiFe] active center [23].

Theoretical [24], [25] and experimental studies [26] attempted to identify the residues involved in the proton translocation. Thus, biochemical and biophysical analysis of the Glu18Gln mutant of the *D. fructosovorans* enzyme (*Desulfovibrio gigas* numbering; Glu18 corresponds to Glu25 in *D. fructosovorans*) indicated the essential role of the conserved Glu18 in proton transfer, as inferred from the crystallographic data [26]. According to another suggestion, which is based on structural analysis of various [NiFe] hydrogenases, the proton transfer pathway involves Arg463, Asp528, His108 and Arg404 (residue numbers refers to the large subunit of *D. gigas* enzyme) [18].

**Figure 1:**
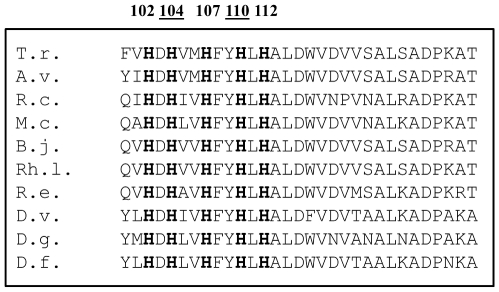
Arrangement of conserved histidines. T.r.: *Thiocapsa roseopersicina* HynL, A.v.: *Allochromatium vinosum* HydL, R.c.: *Rhodobacter capsulatus* HupL, M.c.: *Methylococcus capsulatus*, B.j.: *Bradyrhizobium japonicum* HupL, Rh.l.: *Rhizobium leguminosarum* HupL, R.e.: *Ralstonia eutropha* H16 HoxG, D.v.: *Desulfovibrio vulgaris* Miyazaki F HynB, D.g.: *Desulfovibrio gigas* HynB, D.f.: *Desulfovibrio fructosovorans* HynB. The numbering refers to the *T. roseopersicina* HynL subunit.

We have noted that the sequence alignment of the large subunits of almost all membrane-bound [NiFe] hydrogenases known today shows a highly conserved, symmetrically arranged histidine-rich region: HxHxxHxxHxH (Fig. 1). However, only the second and the fourth histidines are present in the cytoplasmic [NiFe] hydrogenases. A conserved sequence element within this motif was pointed out previously as one of the five consensus motifs present in the large subunits of [NiFe] hydrogenases: (L1) RGxE, (L2) RxCGxCx_3_H, *(L3) Hx_6_L*, (L4) Gx_4_PRGx_3_H, and (L5) DPCx_2_Cx_2_H/R (see also Fig. 1) [27]. These histidines are in the proximal environment of the Ni atom. Chemical modification of internal histidine residues (HxxHxxH) has been carried out on *Desulfovibrio desulfuricans* Norway [NiFeSe] hydrogenase and these residues have been suggested to be implicated in the catalytic process [28]. Burgdorf and co-workers also investigated the L3 motif and introduced point mutations in the HoxH subunit of the soluble hydrogenase (SH) of *R. eutropha*, although they examined only the Leu118 of the L3 motif [29]. When Leu118 was replaced by Ile or Ala, the activities were around 50–70% of the wild type enzyme, changing Leu118 to Phe resulted in a more pronounced (≈75%) activity loss.

One of the aims of this work was to elucidate the function of this His-rich region. The model enzyme in these experiments was the HynSL hydrogenase of the *T. roseopersicina*, which can be selectively mutated, expressed and analyzed in its homologous host. It is shown that the second histidine, which is conserved in both cytoplasmic and membrane associated [NiFe] hydrogenases, has crucial role in the enzyme activity. Based on computational modeling and experimental results, this histidine appears to be part of a strongly hydrogen bonded molecular chain between the active site and the protein surface. It is suggested that these residues might serve as an alternative proton translocation route within the enzyme. In order to test this assumption amino acid substitutions were carried out and it was demonstrated that the mutation of Arg487, Asp103, His104 and Glu436 (Arg479, Asp123, His124, Glu433 in *Desulfovibrio vulgaris*) influence the activity of the HynSL during the catalytic cycle of the enzyme corroborating the proton transfer pathway hypothesis.

## Results

### Functional analysis of the His-rich region in the HynL subunit of T. roseopersicina [NiFe] hydrogenase by site-directed mutagenesis

A highly conserved histidine-rich region was recognized in the large subunit of [NiFe] hydrogenases. The HxHxxHxxHxH sequence typically occurs in the large subunit of most membrane-bound hydrogenases, and, remarkably, two of these conserved histidines are present in the cytoplasmic ones (Fig. 1). We have used the HynSL from *T. roseopersicina* as model enzyme to examine the possible functions of the specific histidine motif.

Various mutant strains were generated in the *hynL* gene by site-directed point mutagenesis. Alanine has been chosen to replace each conserved histidine residue individually and in various combinations. Histidine is a polar, hydrophilic amino acid harboring an imidazol group, accordingly it can be positively charged. On the contrary, alanine is a non-polar, hydrophobic residue so this amino acid is not supposed to be able to fulfill the original role of the histidine irrespective of the nature of this function. As a first approach, single amino acid mutants were generated where the individual conserved histidines were replaced by alanine (Table 1). Thus MH102A, MH104A, MH107A, MH110A and MH112A mutants (Table 2) were generated where the numbers mark the positions of the histidines in the *T. roseopersicina* HynL primary sequence.

**Table 1:** Vectors used in this study.

Vectors	Relevant genotype	Reference or source
pBluescript SK (+)	Cloning vector, Amp^r^	Stratagene
pTHOE5M	*hynS-isp1-isp2-hynL* operon is pDSK509 vector	B. D. Fodor, unpublished
pMH102A	pTHOE5M carrying H102A mutation in *hynL*	This study
pMH104A	pTHOE5M carrying H104A mutation in *hynL*	This study
pMH104F	pTHOE5M carrying H104F mutation in *hynL*	This study
pMH07A	pTHOE5M carrying H107A mutation in *hynL*	This study
pMH110A	pTHOE5M carrying H110A mutation in *hynL*	This study
pMH112A	pTHOE5M carrying H112A mutation in *hynL*	This study
pM102/104/107/110/112A	pTHOE5M carrying penta-His mutations in *hynL*	This study
pMH102/107/110/112A	pTHOE5M carrying tetra-His mutations in *hynL*	This study
pMR487I	pTHOE5M carrying R487I mutation in *hynL*	This study
pMD103L	pTHOE5M carrying D103L mutation in *hynL*	This study
pME436I	pTHOE5M carrying E436I mutation in *hynL*	This study
pME14Q	pTHOE5M carrying E14Q mutation in *hynL*	This study

The plasmid derivatives (pMH102A, pMH104A, pMH107A, pMH110A, pMH112A) (Table 1) were transferred into *T. roseopersicina* GB112131 strain (Table 2) by conjugation. The mutant HynSL strains expressed the enzyme on a background deficient of all other [NiFe] hydrogenases of *T. roseopersicina*, hence the effects of each single amino acid mutation could be examined in Hyn activity assays both *in vivo* and *in vitro*.

**Table 2:** Bacterial strains.

Strain/plasmid	Relevant characteristics	Reference or source
*T. roseopersicina*		
BBS	wild type	[4]
GB112131	*hynSL*::Sm^r^ *hupSL*::Gm^r^ *hoxH*::Em^r^ ,(Em^r^) oriented as *hox* operon	[30]
MH102A	pMH102A plasmid in GB112131 strain	This study
MH104A	pMH104A plasmid in GB112131 strain	This study
MH104F	pMH104F plasmid in GB112131 strain	This study
MH107A	pMH107A plasmid in GB112131 strain	This study
MH110A	pMH110A plasmid in GB112131 strain	This study
MH112A	pMH112A plasmid in GB112131 strain	This study
MH102/104/107/110/112A	pMH102/104/107/110/112A plasmid in GB112131 strain	This study
MH102/107/110/112A	pMH102/107/110/112A plasmid in GB112131 strain	This study
MR487I	pMR487I plasmid in GB112131 strain	This study
MD103L	pMD103L plasmid in GB112131 strain	This study
ME436I	pME436I plasmid in GB112131 strain	This study
ME14Q	pME14Q plasmid in GB112131 strain	This study
*E. coli*		
S17-1(λpir)	294 *(recA pro res mod)* Tp^r^, Sm^r^ (pRP4-2-Tc::Mu-Km::Tn7), λ*pir*	[44]
XL1-Blue MRF′	Δ(*mcrA*)183, Δ(*mcrCB-hsdSMR-mrr*)173, *endA1*, *supE44*, *thi-1*, *recA1*, *gyrA96*, *relA1 lac* [F′ *proAB lacI^q^Z*ΔM15 Tn10 (Tc^r^)]^c^	Stratagene

Indicated strains and plasmids are from Stratagene, La Jolla, CA, USA.

The *in vivo* hydrogen evolving capacity of the single amino acid mutants was compared to the wild type HynSL (GB112131+pTHOE5M strain). The single amino acid mutants and the control strains were grown anaerobically in standard Pfennig medium for 6 days and the accumulated H_2_ was measured by gas chromatograph. The growth rates of the mutants and the controls were very similar (the optical density values of the cultures were continuously measured at 600 nm; data not shown), and the strains started to evolve H_2_ nearly at the same time. *In vivo* H_2_-production (using endogenous electron donors) was significantly altered only in the case of MH104A mutant strain (Table 3), i.e., 40% of the wild type activity could only be measured. In the experiments GB112131 served as negative control since this strain does not express Hyn, Hup and Hox1 enzyme (Δ*hynSL*, Δ*hupSL*, Δ*hox1H*) [30] and under the conditions used Hox2 is not active [6]. The *in vivo* hydrogen evolution of MH102A, MH107A, MH110A, MH112A single amino acid mutants did not change remarkably (Table 3).

**Table 3:** *In vivo* and *in vitro* hydrogenase activity of *Thiocapsa roseopersicina* HynSL enzyme.

Strain	*In vivo* H_2_ production (%)	*In vitro* H_2_ production (%)	*In vitro* H_2_ uptake (%)
GB112131	0	0	0
GB112131+pTHOE5M	100.0	100.0	100.0
MH102A	91.8±20.8	64.5±16.8	67.7±7.4
MH104A	39.5±8.0	6.1±5.2	18.2±13.7
MH107A	88.9±15.2	78.6±3.6	85.6±6.9
MH110A	71.4±7.4	73.5±8.7	95.2±6.6
MH112A	81.8±22.0	65.9±13.3	76.8±18.4
MH102/104/107/110/112A	32.5±0.0	19.5±7.3	32.2±18.4
MH102/107/110/112A	57.7±4.4	63.7±10.3	85.1±12.5
MR487I	0	0	0
MD103L	0	0	0
MH104F	43.2±16.8	0	5.5±2.8
ME436I	29.68±12.4	50.9±7.8	54.58±13.2
ME14Q	0	51.2±5.6	46.81±3.8

*In vitro* hydrogenase activity of the mutant and control strains was also assayed after 6 days of growth. The H_2_ uptake activity was not or slightly altered in case of MH102A, MH107A, MH110A or MH112A mutants compared to the wild type, but it was reduced substantially when the 104^th^ His residue of HynL was replaced by alanine (Table 3). Therefore, it was concluded that the low *in vivo* hydrogen producing capability was the consequence of reduced *in vitro* hydrogenase activity of the mutant strain. It can be observed that the *in vitro* and the *in vivo* activity dropped to about 6% and 40% of the wild type enzyme, respectively. These data can be interpreted by assuming that the Hyn hydrogenase is in excess in the wild type cells and its amount is not the bottleneck of the *in vivo* hydrogen evolution. Therefore, a slight reduction in the Hyn activity (*in vitro*) might be enough for converting all substrates - available for the enzymes in the cells - to products. In our case, the remaining 6% activity can convert approximately 40% of the total electron/proton flux toward the Hyn enzyme in the cells.

In order to get information on the localization of the mutant enzymes, *in vitro* hydrogen uptake activities were measured in the membrane and soluble fractions. The results clearly showed that the mutations did not change membrane-Hyn hydrogenase interaction (data not shown).

### Characterization of multiple His mutations

In order to get deeper insight into the role of the His-rich motif in enzyme activity, two additional mutants were created. In the penta-His mutant, alternatively referred to as MH102/104/107/110/112A, all five conserved His were replaced by Ala. In the tetra-His mutant strain, the construct harbored only one of the conserved His (His104), while the other four His were substituted by Ala (MH102/107/110/112A) (Table 2). The *in vivo* and *in vitro* hydrogenase activities of the MH102/104/107/110/112A (penta-His mutant) strain were very similar to those of the single amino acid mutant strain, MH104A, while in case of the MH102/107/110/112A tetra-His mutant, where His104 remained intact, the effect of the replacement of all four histidines (102/107/110/112) had significantly less effect on the enzyme activities than the His104 mutation alone (Table 3).

### Computational modeling of the hydrogen bond network

Hydrogen bond networks probably play an essential role in understanding certain aspects of how hydrogenases function via the Grotthuss mechanism. Protein residues and water molecules participating in this mechanism form the so-called proton translocation routes of the hydrogenases. The importance of hydrogen atoms in proton pathways might be relevant.

In order to get deeper insight at the molecular level into the functionality of the conserved His residues studied, the analysis of the 3-dimensional (3D) structure is necessary. However, no experimental 3D structure has been published yet for the HynSL enzyme of *T. roseopersicina*. Note that a homology model [31] has been established based on multi-structural alignments where the structure of the *Desulfovibrio gigas* hydrogenase (PDB ID: 2FRV) was used as a template. This homology model of HynSL cannot be used to propose possible proton-hopping mechanisms due to the fact that positions of structural water molecules are unknown.

Another possibility is to analyze a 3D structure of a related hydrogenase which contains conserved residues in the relevant protein region (around the five conserved His in the case of HynSL) and in which at least the atomic positions of the water oxygens are known. Based on this reasoning the high resolution (1.50 Å) X-ray structure of the reduced form of [NiFe] hydrogenase of *D. vulgaris* Miyazaki F (PDB ID: 1WUL [32]) has been chosen as a model of the large subunit for structural analysis. In order to confirm the model choice, the structures of the homology modelled HynL and experimentally determined HynB were compared and their structural alignment revealed that the His motifs studied are structurally conserved in these proteins (Fig. S1). Therefore, the protonation microstates and hydrogen bond network of the [NiFe] hydrogenase of *D. vulgaris* Miyazaki F was modeled as described in “Materials and Methods”.

**Figure 2:**
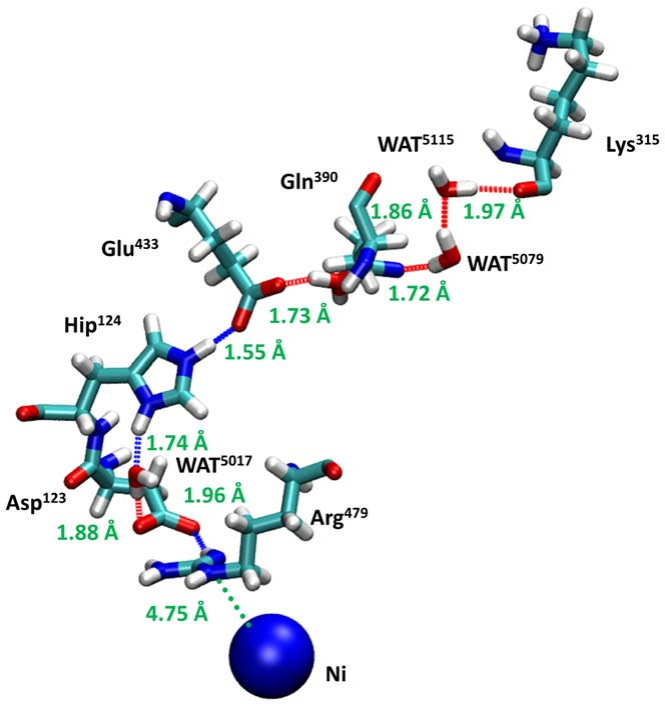
The proposed proton transfer pathway based on the structure of *Desulfovibrio vulgaris* Miyazaki F. (PDB: 1WUL). Cyan, blue, red and white colors represent carbon, nitrogen, oxygen and hydrogen atoms, respectively except blue sphere which stands for nickel atom. Distances of hydrogen bonding partner are given in Angstroms.

**Figure 3:**
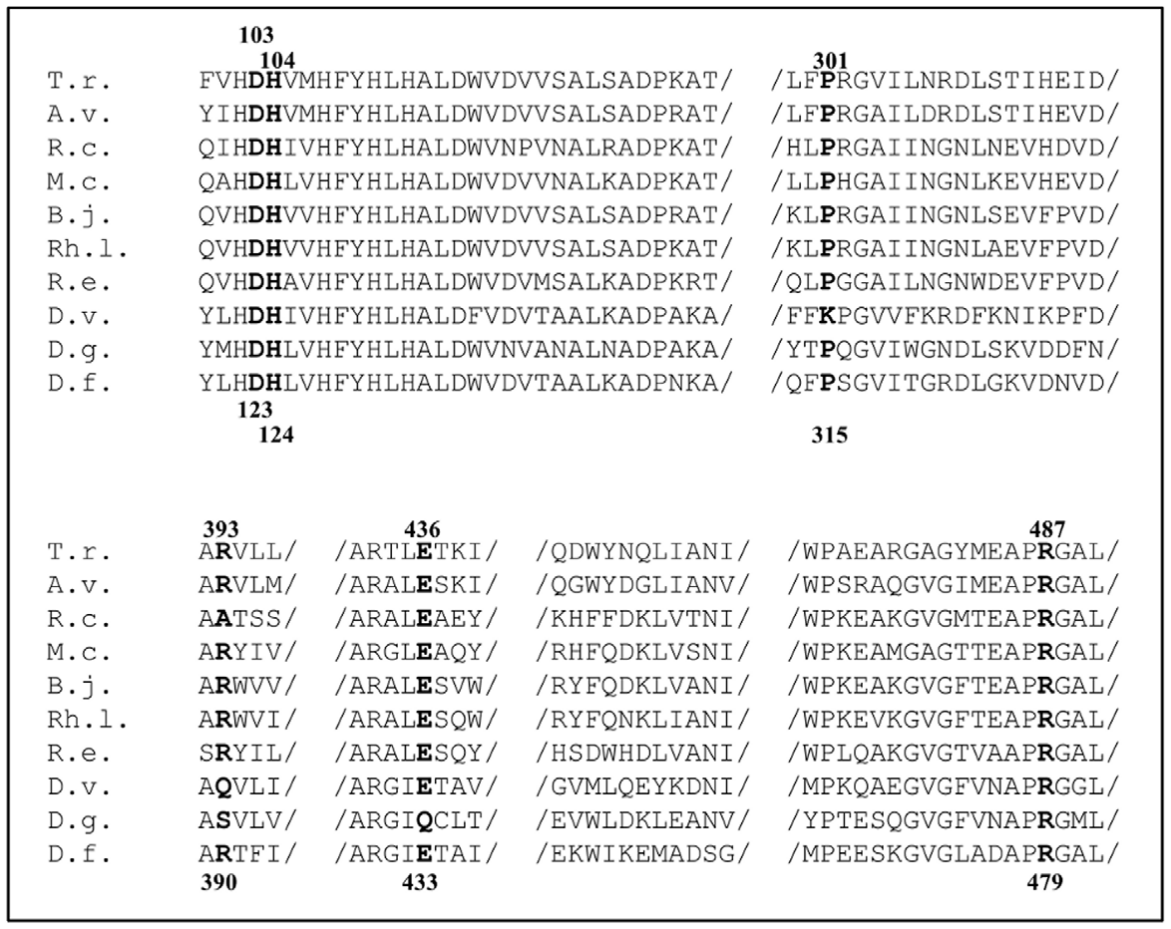
The amino acids of proposed proton transfer pathway are highly conserved. T.r.: *Thiocapsa roseopersicina* HynL, A.v.: *Allochromatium vinosum* HydL, R.c.: *Rhodobacter capsulatus* HupL, M.c.: *Methylococcus capsulatus*, B.j.: *Bradyrhizobium japonicum* HupL, Rh.l.: *Rhizobium leguminosarum* HupL, R.e.: *Ralstonia eutropha* H16 HoxG, D.v.: *Desulfovibrio vulgaris* Miyazaki F HynB, D.g.: *Desulfovibrio gigas* HynB, D.f.: *Desulfovibrio fructosovorans* HynB. The upper and lower numbering refers to the large subunits of *T. roseopersicina* and *D. vulgaris* enzymes, respectively.

In the active site of the hydrogenase, a positively charged nickel is coordinated by cysteines and next to this complex a neutral conserved Arg479 (Arg487 in *T. roseopersicina*) is situated at a distance of 4.75 Å (Fig. 2). This unusual protonation state of Arg479 can be interpreted as a consequence of the close proximity of the positively charged Ni. Asp123 (Asp103 in *T. roseopersicina*) and Arg479 form a strong ion-pair-like structure as indicated by the short distance between O@Asp123 and H@Arg479 (1.96 Å). Asp123 also interacts with a water molecule connecting its side chain to the next residue (Hip124; Hip104 in *T. roseopersicina*) via strong hydrogen bonds (It should be noted that histidine with hydrogen on the epsilon nitrogen assigned as Hie, while Hip indicates histidine with hydrogens on both nitrogens.). Our calculation reveals that this residue is protonated and positively charged which is unique among the highly conserved histidines studied in this work. Due to the enhanced possibility for hydrogen bonding, the other hydrogen of Hip124 (at epsilon position) binds tightly to the oxygen of Glu433 (Glu436 in *T. roseopersicina*) (1.55 Å). Again, the short distance (1.73 Å) between the glutamic acid (Glu433) and glutamine (Gln390) can be a manifestation of a strong interaction. Finally, side chains of Gln390 and Lys315 are hydrogen bonded through a water dimer (Arg393 and Pro301 can be found in the latter positions in *T. roseopersicina*). The Lys315 is located at the hydrogenase surface. The above mentioned network of strong hydrogen bonds may be a good candidate where the Grotthuss mechanism can take place. It is also important to emphasize that elements of this network (Arg479, Asp123, Hip124 and Glu433 residues, *D. vulgaris* numbering) are found to be highly conserved in all membrane-bound hydrogenases (Fig. 3). The structural alignment of the *D. vulgaris* and *T. roseopersicina* hydrogenase revealed that the positions of these residues are structurally conserved, as well (see Fig. S2). In contrast to this extended hydrogen bond network involving Hip124, remaining conserved histidines (Hie122, Hie127, Hie130 and Hie132 in *D. vulgaris*; Hie102, Hie107, Hie110, Hie112 in *T. roseopersicina*) are not only neutral, but their H-bond networks are far more limited.

Recently, crystal structure of the [NiFe] hydrogenase from *Allochromatium vinosum* (PDB ID: 3MYR [16]) was solved and published in the PDB database. Although this enzyme shows higher sequence homology to the hydrogenase of *T. roseopersicina* relative to that of *D. vulgaris* Miyazaki F, its 3D resolution is not as detailed as in the case of *D. vulgaris*. After analyzing the structure of hydrogenase from *A. vinosum*, we could draw the same conclusion: very similar hydrogen bonding network has been identified including Arg487-Asp103-WAT3408-His104-Glu436 (according to the numbering of the primary sequence of *A. vinosum*; the residue ID of the water molecule is 3408 in the 3MYR structure). It is important to note that the position of the oxygen atom of the structural water along this proton transfer pathway is also conserved based on the three dimensional structure of the hydrogenases of *D. vulgaris* and *A. vinosum*. Further elements of the proton transfer pathway consist of polar non-conserved residues and structural water (Glu436-Arg393-Glu484-WAT; where the numbering of the water molecule is 3505 in the 3MYR structure) which may be covered by a flexible loop region of the hydrogenase enzyme of *A. vinosum*.

### Experimental validation of the computational model

In order to test the model based on computational analysis of 3D hydrogenase structures, the first four amino acids of the proposed hydrogen bonded chain (Arg487, Asp103, His104 and Glu436 *T. roseopersicina* numbering and Arg479, Asp123, His124, Glu433 *D. vulgaris numbering*) were mutated, since the rest of the amino acids of this chain seemed to be not conserved in the membrane-associated hydrogenases. Arg487 located in the vicinity of the active site and His104, being within the hydrogen bond distance from the other members of the discussed hydrogen bonded chain, were suggested formerly as part of a possible proton transfer pathway [18]. By exchanging the large subunit amino acids in this network with non-protonable but sterically similar ones we experimentally tested the assumed proton channel. Arg487 and Glu436 were replaced by Ile, Asp103 was substituted by Leu, His104 by Phe, and the activity of the mutants was compared to that of the wild type enzyme. Since Glu14 (Glu25 in *D. fructosovorans*) was previously shown as proton transfer gate during the catalytic cycle of the [NiFe] hydrogenases [26], an additional mutant, the Glu14Gln was also made in HynL to validate this observation in *T. roseopersicina*.

The *in vitro* hydrogen uptake and the hydrogen evolving capacities of the mutant enzymes were measured using redox viologen dyes, as described before. In the MR487I, MD103L the *in vitro* hydrogenase activity was totally lost, while in case of ME436I, the HynSL enzyme activity was approx. 50% of the wild type (Table 3). In case of MH104F mutant the *in vitro* H_2_ uptake activity of the enzyme was strongly decreased (about 6% of the wild type enzyme activity which coincides with the values obtained for the MH104A mutant) and the *in vitro* H_2_ production of the mutant was practically zero.

### Posttranslational maturation of the mutants

**Figure 4:**
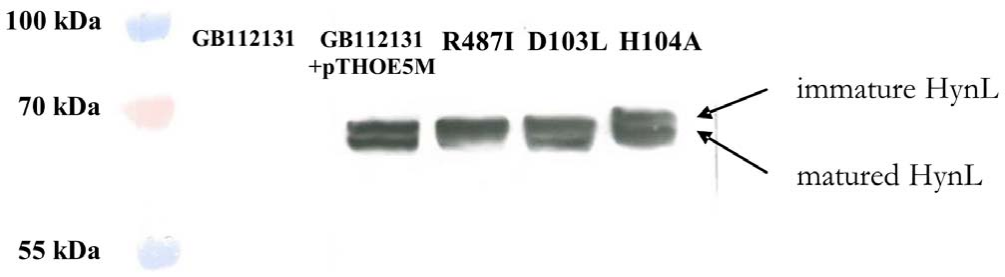
Western-analysis of amino acid mutant strains. Amino acids implicated in the proposed proton transfer pathway were replaced by site-directed mutagenesis. HynL was detected using Anti-HupL antibody, 25 µg of total proteins were applied both for the controls and for the mutant strains. GB112131: *T. roseopersicina ΔhynSL*, *ΔhupSL*, *ΔhoxH1* strain, the negative control; GB112131+pTHOE5M: *T. roseopersicina ΔhynSL*, *ΔhupSL*, *ΔhoxH1* strain+wild type HynSL expressing vector, pTHOE5M, the positive control. Mutated strains were the follows: R487I: *T. roseopersicina* MR487I strain, D103L: *T. roseopersicina* MD103L strain, H104A: *T. roseopersicina* MH104A strain.

In order to confirm that the activity change observed in the MH104A mutant strain reflected the reduced specific activity of the mutant enzyme and was not due to a failure in its biosynthesis or proteolytic instability, Western-hybridization experiments were carried out (Fig. 4.). Since, the *in vitro* hydrogenase activities of the MH104A and the MH104F mutants seemed practically the same, only the MH104A mutant was used for Western-hybridization experiments. Both the immature and the fully matured protein bands could be observed in the MD103L and MH104A mutants as well as the positive control and could clearly be distinguished from each other by their different migration properties. In the MR487I mutant only the immature form was detectable. Since the proteolytic cleavage occurs only when the metal center is assembled and inserted, the appearance of band corresponding to the mature enzyme means the presence of the NiFe center in these protein forms. Comparison of the band intensities of the MD103L, MH104A mutants and the control enzyme indicated the presence of the approximately same amount of both HynL forms. Thus the strongly reduced activity of the MH104A mutant hydrogenase could not be attributed to proteolysis or decreased biosynthesis of the enzyme. However, in the case of R487I mutant, the lack of the mature large subunit indicates that substitution of this residue interferes with the maturation process of the HynL. This does not exclude the proton transfer role of Arg487 in the catalytic processes.

### Testing other route: Glu14 is not essential in *T. roseopersicina*

An extensive previous study has demonstrated that the Glu18 of *D. fructosovorans* hydrogenase (Glu14 in *T. roseopersicina*) has essential role in proton translocation processes [26]. In order to test the role of this residue in *T. roseopersicina*, it was replaced by Gln and the effect of this mutation was compared with the results obtained for similar *D. fructosovorans* Glu18 mutants [26]. The *in vitro* hydrogenase activity of the mutant *T. roseopersicina* HynSL was about 50% of the wild type hydrogenase activity and was not diminished totally as in the Glu18Gln *D. fructosovorans* mutant. The negative results obtained for the *in vivo* H_2_ evolution of ME14Q is quite surprising. Since, we could detect both uptake and evolution activity of the enzyme *in vitro*, and their relative values were similar, the lack of *in vivo* H_2_ evolution would indicate that the functional interaction of the enzyme with the other cellular processes is somehow blocked. The clarification of the phenomenon definitely needs further studies but it does not overthrow the model suggested.

## Discussion

Sequence comparison of [NiFe] hydrogenases revealed at least five consensus motifs in the large subunits of [NiFe] hydrogenases: L1–L5 [27]. In addition there are highly conserved regions and conserved amino acids typical for [NiFe] hydrogenases, which have important roles for example in coordination of the metals (Mg ion) [33], electron transfer [11], [21], proton transfer [26], gas access to or from the active site, O_2_ insensitivity [17], [34]–[38] or in the interaction with protein partners [2].

The L3 motif (Hx_6_L) is composed of amino acids being in the vicinity of the metal center. This motif overlaps with the histidine-rich region analyzed in this study.

### Possible roles of the His-rich region

The HxHxxHxxHxH motif (major part of the L3 motif) in the 100–120 amino acid region of the large subunits is highly conserved in the various membrane-bound hydrogenases. Sequence alignment of [NiFe] hydrogenases revealed that only two of these conserved histidines are present in the cytoplasmic hydrogenases (His104 and His110, in *T. roseopersicina* corresponds to His124, His130 in *D. vulgaris*). In this work various mutant strains were generated in the *hynL* gene by site-directed point mutagenesis in order to disclose the real function of the histidines of this conserved motif. Based on the *in vivo* and *in vitro* hydrogenase activity measurements of single and multiple amino acid mutants it was concluded that only the His104 (His124 in *D. vulgaris*) has important role in the enzymatic function of the HynSL enzyme. The next question was how the sole His104 could be involved in the enzyme activity while the other histidines comprising the His motif did not alter the enzyme activity so much.

Based on the results of hydrogenase activity measurements on His mutants, several suggestions can be made regarding the function of His104. Apparently its distance from the active center seems to be too large for direct interaction with the binuclear metal center (Table 4). Histidines might have various functions in proteins including electron or charge transfer, structure stabilization via ionic interaction or liganding metals/redox cofactors. In [NiFe] hydrogenases of *Desulfovibrio* species, electrons are transferred from the active site to the redox partner via a proximal [4Fe4S], a medial [3Fe4S], and a distal [4Fe4S] cluster. The membrane-bound periplasmic hydrogenases are complexed to cytochrome b (as electron acceptor), allowing electron transfer to a quinone [18]. However, the histidine cluster of interest is located far from the proposed electron channel of the protein (on the opposite side), thereby it is unlikely that these amino acids play role in the electron transfer within the hydrogenases. Histidines are also good candidate for being part of proton transfer in redox enzymes [39], since relatively small shifts in pH change its protonation microstate. Several proton transfer pathways have been proposed for [NiFe] hydrogenases, based on the respective three-dimensional structures, and proton transport during catalysis probably does not use a single route [40]. One of the first proposed paths included a glutamate, four histidines and some water molecules [11]. In this proton transfer route, the first acceptor of the proton is His72 from the large subunit, which would receive the proton from a bridging Cys533. Later, it was shown in *D. fructosovorans* hydrogenase that the mutation of the Glu18 to an Asp decreases considerably the activity of the hydrogenase, and mutation of Glu18 to a Gln completely abolished it [26]. Consequently, Glu18 is likely to be part of the proton transfer pathway. Several theoretical studies were performed to confirm this Glu18 related route and to identify another possible proton pathways [18], [24], [25]. All these proposed routes were described using 3D structure of [NiFe] hydrogenases belonging to sulfate-reducing bacteria of *Desulfovibrio* genus. According to one of the proposed pathways [18], first the proton is translocated to the terminal cysteine Cys530, and continues with Glu18, several water molecules, Glu46 and water molecule coordinating the Mg atom. However, changing the Glu14 in the HynL subunit (corresponding to Glu18 in *D. gigas*.) revealed its less pronounced role in the *T. roseopersicina* hydrogenase and raised the possibility of alternative proton translocation route(s). Accordingly, in the above-mentioned paper, another model suggests that amino acids of the large subunit of *D. gigas*, namely the Arg463 (Arg487 in *T. roseopersicina*), Asp528, His108 (His104 in *T. roseopersicina*) and Arg404 can be part of a proton pathway [18].

**Table 4:** Distance of active site Ni atom and the center of mass (COM) of the conserved histidine amino acids of *Desulfovibrio vulgaris* Miyazaki F.

Conserved amino acids of *Desulfovibrio vulgaris* Miyazaki F	d(Ni-COM) (Å)^b^
His 122^a^	12.00
His 124^a^	11.69
His 127^a^	11.46
His 130^a^	15.52
His 132^a^	15.85

a His122, His124, His127, His130, His132 in *Desulfovibrio vulgaris* Miyazaki F correspond to His102, His104, His107, His110, His112 in *T. roseopersicina*.

b The center of mass is the mean location of the mass in an amino acid residue.

### The alternative proton translocation pathway

In order to clarify the role of this residue, molecular modeling calculations were carried out and a network of conserved amino acids connected by strong hydrogen bonds was recognized. Moreover, the amino acids being part of this network are highly conserved among the membrane-associated [NiFe] hydrogenases (Fig. 3 and Fig. S2). Therefore, it was concluded that this molecular chain - with the participation of His104 - might be a good candidate for a proton transfer pathway where the Grotthuss mechanism can take place. In contrast to this extended hydrogen bond network the other conserved His residues of the L3 motif (His102, His107, His110, His112 in *T. roseopersicina*, and His122, His127, His130, His132 in *D. vulgaris*, respectively) are not only neutral, but their H-bond network is far more limited. Remarkably His104 is one of the two conserved histidines of the analyzed motif (His110 is the other), which occurs in the large subunits of the cytoplasmic [NiFe] hydrogenases as well. This corroborates the importance of His104 in all [NiFe] hydrogenases known so far regardless of their cellular location. A previous review [18] already suggested a pathway which implicated Arg463 and His108 (corresponding to Arg487 and His104 in *T. roseopersicina* HynL), the two conserved amino acids which are also members of the molecular chain proposed in this article. This route includes other histidines of the analyzed L3 motif (His107 and His112, *T. roseopersicina* numbering) as well. However, our experimental data showed that the mutation of the His107 and His112 did not alter the activity of the hydrogenase, thus it is slightly presumptive that these amino acids could be part of a proton transfer pathway. The additional residues of the potential proton pathway are basically different from our suggestion, thus we propose a modification of the previously suggested route and provide an alternative possibility how protons can be transferred between the active site and the surface of the enzyme.

In order to verify the proposed hydrogen bond network as an alternative proton transfer pathway additional amino acid mutants were constructed. We have focused mainly on the first three amino acids of the proposed hydrogen bond network (Arg487, Asp103, His104 in *T. roseopersicina*, and Arg479, Asp123, His124 in *D. vulgaris* respectively) since Glu436 (Glu433 in *D. vulgaris*) is not conserved in all membrane-bound [NiFe] hydrogenases (e.g. in *D. gigas* and *D. desulfuricans* in the appropriate site of the hydrogenase large subunit there is Gln instead of Glu), and Glu436 stands after the His104 in this proposed pathway, where His104 could be a diverge point in different possible proton transfer routes due to the adjacent water molecules. Additionally, based on the analysis of available 3D hydrogenase structures, it was recognized that even in previously described proton transfer pathways only the amino acids in the proximity of the Ni are highly conserved among [NiFe] hydrogenases of different species. Furthermore the consistence of the pathways near by the surface of the protein is less extended.

It was clearly demonstrated that Arg487 had essential role in the formation (and likely in the function) of the active enzyme. Asp103 was also indispensable for the activity, nevertheless its substitution did not block the maturation processes. His104 was still very important: its replacement did not abolish but dramatically reduced the activity of HynSL. Substitution of other residues in the model revealed their less critical role in the enzymatic processes. Arg487 might have dual role: since its lack blocked the maturation processes and - according to the *in silico* analysis - it might be also involved in the proton translocation processes. However, matured large subunit could be synthesized in all other cases, the mutations have changed the hydrogenase activity of the posttranslationally processed HynSL. The coordination of the Ni and the Fe in the metal center of hydrogenases is achieved by the well-characterized CxxC motifs but the highly conserved Arg487 may have also important, indirect role in this process, since it can establish two hydrogen bonds with CN^−^ ligands with the active site Fe [41], thus might have influence on the catalytic cycle of [NiFe] hydrogenase.

In this article we mapped the possible function of a large subunit His-rich motif of the membrane associated [NiFe] hydrogenases and demonstrated that one member of this motif, His104, has important role in the activity of the enzyme and could be part of an alternative proton transfer route. Impairing the protein environment of active site through mutation of conserved amino acids in *T. roseopersicina* HynL coincides with the conclusions from computational modeling that a hydrogen bond network, with the participation of His104, can function as a proton transfer channel during the catalytic cycle of the [NiFe] hydrogenases. This is the first study where an *in silico* predicted proton translocation pathway was systematically verified by site-directed mutagenesis in [NiFe] hydrogenases.

An interesting feature of this model compared to the previous suggestions is that this proton channel is located on the opposite side of the large subunit relative to the position of the small subunit. It should be emphasized that this is the first time when the conclusions of a molecular modeling study could be checked against a crystal structure distinct from the well-known *Desulfovibrio*-type. The general features of the proposed novel proton channel could be detected in the *A. vinosum* structure as well.

## Materials and Methods

### Bacterial strains and plasmids

Plasmids and strains are listed in Table 1 and Table 2, respectively. *T. roseopersicina* strains were grown photoautotrophically in Pfennig's mineral medium under anaerobic conditions in liquid cultures with continuous illumination (Philips 60W Golf Ball ES Clear Bulbs, 640 lm, average photon flux at the sample was 64.5 µmol s^−1^·m^−2^) at 27–30°C for 5–6 days [4], [6]. Plates were supplemented with acetate (2 g l^−1^) and solidified with Phytagel (7 g l^−1^) (Sigma, St Louis, MO, USA) [42]. The plates were incubated in anaerobic jars of the AnaeroCult (Merck, Darmstadt, Germany) system for two weeks. *E. coli* strains were maintained on LB-agar plates. Antibiotics were used in the following concentrations (µg ml^−1^): for *E. coli*: ampicillin (100), kanamycin (25); for *T. roseopersicina*: gentamicin (5), kanamycin (25), streptomycin (5), erythromycin (50).

### Conjugation

Conjugation was carried out as described previously [43].

### Identification of His-rich region

Protein sequence comparisons were done with the BLASTP (http://pbil.univ-lyon1.fr/BLAST/blast_prot.html) and CLUSTAL X software.

### Site-directed mutagenesis

The QuikChange XL site-directed mutagenesis kit (Stratagene, Amsterdam, The Netherlands) was used to generate point mutations in the large subunit gene *hynL*. The ApaI fragment from pTHOE5M vector (including the entire *hyn* operon) was cloned into pBluescriptSK+ to generate pBtHynLApaI that was used as a template in mutagenesis experiments, except in the experiment generating the tetra-His mutant. In the latter case the plasmid already containing the H102A mutation was used as a template in the PCR reaction. After mutagenesis, the ApaI fragment was sequenced and inserted back into the ApaI-digested pTHOE5M. The recombinant plasmid was introduced first into *E. coli* S17/1 [44] by chemical transformation then conjugated into *T. roseopersicina* GB112131 strain. The primers used for the mutagenesis are listed in Table 5.

**Table 5:** Mutagenesis primers used for creating new mutants.

Mutagenesis primers	
OH102AF	5′-GATCAGCTCGCAGTTCGTG**GCA**GATCATGTGATGCACTTCTAT-3′
OH102AR	5′-ATAGAAGTGCATCACATGATC**TGC**CACGAACTGCGAGCTGATC-3′
OH104AF	5′-CGCAGTTCGTGCACGAT**GCA**GTGATGCACTTCTATCACC-3′
OH104AR	5′-GGTGATAGAAGTGCATCAC**TGC**ATCGTGCACGAACTGCG-3′
OH107AF	5′-GTGCACGATCATGTGATG**GCA**TTCTATCACCTGCACGCG-3′
OH107AR	5′-CGCGTGCAGGTGATAGAA**TGC**CATCACATGATCGTGCAC-3′
OH110AF	5′-GTGATGCACTTCTAT**GCC**CTGCACGCGCTCG-3′
OH110AR	5′-CGAGCGCGTGCAG**GGC**ATAGAAGTGCATCAC-3′
OH112AF	5′-CACTTCTATCACCTG**GCC**GCGCTCGACTGGG-3′
OH112AR	5′-CCCAGTCGAGCGC**GGC**CAGGTGATAGAAGTG-3′
O4HTOAF	5′-GATCATGTGATG**GCA**TTCTAT**GCC**CTG**GCC**GCGCTCGACTGGG 3′
O4HTOAR	5′-CCCAGTCGAGCGC**GGC**CAG**GGC**ATAGAA**TGC**CATCACATGATC 3′
O5HTOAF	5′-CTCGCAGTTCGTG**GCA**GAT**GCA**GTGATG**GCA**TTCTAT**GCC**CTG**GCC**GCGCTC GACTGGG-3′
O5HTOAR	5′-CCCAGTCGAGCGC**GGC**CAG**GGC**ATAGAA**TGC**CATCAC**TGC**ATC**TGC**CAC GAACTGCGAG-3′
R487IF	5′-GTTACATGGAGGCCCCG**ATC**GGCGCGCTCGGTCACTGG-3′
R487IR	5′-CCAGTGACCGAGCGCGCC**GAT**CGGGGCCTCCATGTAAC-3′
D103LF	5′-AGCTCGCAGTTCGTGCAC**CTG**CATGTGATGCACTTC-3′
D103LR	5′-GAAGTGCATCACATG**CAG**GTGCACGAACTGCGAGCT-3′
H104FF	5′-CGCAGTTCGTGCACGAT**TTC**GTGATGCACTTCTATC-3′
H104FR	5′-GATAGAAGTGCATCAC**GAA**ATCGTGCACGAACTGCG-3′
E436IF	5′-GCGGCCCGGACCTTG**ATC**ACGAAGATCCTGACC-3′
E436IR	5′-GGTCAGGATCTTCGT**GAT**CAAGGTCCGGGCCGC-3′
E14QF	5′-ATCCCGTCACCCGTATC**CAG**GGCCATCTGCGCATCG-3′
E14QR	5′-CGATGCGCAGATGGCC**CTG**GATACGGGTGACGGGAT-3′

Nucleotide triplets coding mutated amino acids are bold.

### Activity measurements

The hydrogenase activities of the various mutants were measured both *in vivo* and *in vitro*. In all experiments, the GB112131 strain (*ΔhoxH*, *ΔhupSL*, *ΔhynS-isp1-isp2-hynL*) was used as negative, and the GB112131+pTHOE5M strain (*ΔhoxH*, *ΔhupSL*, *ΔhynS-isp1-isp2-hynL* carrying the *hynSL* operon in pDSK509 vector) as positive control.

### *In vivo* hydrogen evolution measurement

*T. roseopersicina* cultures (60 ml) were grown photochemolithoautotrophically in Pfennig's medium (PC4, 4 g L^−1^ sodium-thiosulfate) under nitrogen atmosphere in sealed 100 ml Hypo-Vial flasks. Anaerobiosis was established by flushing the gas phase with N_2_ for 10 minutes. H_2_ production was followed by injecting 200 µl samples of the headspace into a gas chromatograph (Agilent 6890, TCD detector) on the 6^th^ day of growth.

### Preparation of membrane-associated and soluble protein fractions of *T. roseopersicina*

60 ml of *T. roseopersicina* culture was harvested by centrifugation at 7,000× g for 10 min. The cells were suspended in 1 ml of 20 mM K-phosphate buffer (pH = 7.0), and broken by sonication (Bandelin Sonopuls (Berlin, Germany) HD3100 ultrasonic homogenizer; at 85% amplitude six times for 15 s). The suspension was centrifuged at 10,000× *g* for 15 min at 4°C. The debris (remaining whole cells and sulfur crystals) was discarded and the supernatant (crude extract) was centrifuged at 100,000× *g* for 90 minutes at 4°C. The pellet was washed with K-phosphate buffer (20 mM, pH = 7.0) and used as membrane fraction. The supernatant was considered as the soluble fraction.

### *In vitro* methyl-viologen dependent hydrogen evolution activity measurement

60 ml of *T. roseopersicina* cultures (grown in PC4 medium in sealed 100 ml Hypo-Vials flushed with N_2_) were harvested, crude extract was prepared as described above. The protein content of the extracts were quantified by Micro-Lowry method [45], 0.1 mg ml^−1^ of total protein was used as a sample for the measurement in 20 mM K-phosphate buffer (pH = 7.0) and 40 µl of 40 mM methyl-viologen in final reaction volume of 2 ml. The mixture was flushed with nitrogen for 10 min, the reaction was initiated by injecting 100 µl of anaerobic 50 mg ml^−1^ sodium-dithionite solution. Samples were incubated at 60°C for 1 h, H_2_ content of the gas phase was determined by gas chromatograph.

### *In vitro* hydrogen uptake activity measurement

The samples were resuspended in 2 ml K-phosphate buffer (20 mM, pH = 7.0) then 40 µl of 40 mM benzyl-viologen was added in an anaerobic cuvette. The cuvettes were closed with SubaSeal rubber stoppers. The mixture was flushed with N_2_ for 5 min followed by flushing with 100% H_2_ for another 5 min. Samples were incubated at 60°C in spectrophotometer, rate measurement of hydrogenase activity was performed by following the absorbance at 600 nm.

### Determination of optical density of the cultures

Optical density values of the cultures were measured at 600 nm wavelength by photometer (BIORAD SmartSpec 3000) using 100 µl of the cell culture as a sample. Samples were withdrawn by sterile disposable syringe in order to retain the anaerobic environment. *In vivo* activities were normalized to the optical density.

### Western-hybridization

The crude extracts of the mutants and the control strains grown on standard Pfennig's medium were analyzed by Western-hybridization (preparation and the protein content determination of cell extract is described above). Proteins (50 µg of total protein for the wild type and single His-mutants, 25 µg for the wild type and proton channel amino acid mutants) were separated in 10% SDS–PAGE (running for 3 h at 120 V) and blotted onto nitrocellulose membrane (Whatman) (parameters used: 2 h at 100 V; transfer buffer: 50 ml of 20× Invitrogen Transfer buffer, 100 ml methanol in 1 l buffer). Non-specific binding of proteins were blocked (blocking solution: 5% non-fat milk powder in TBST (500 ml NaCl, 0.05% Tween 20, 10 mM TRIS-HCl pH 7.5). Anti-HupL antibody, kindly provided by Dr. Qing Xu, JCVI, USA, was used as primary antibody at 1∶10000 dilution. The secondary antibody (goat anti rabbit HRP H+L was used at 1∶5000 dilutions in blocking buffer. For detection of the proteins 1-1 ml of enhancer and peroxide solution (Pierce) was used for 1 min and chemiluminescence signal was detected by autography (GE Healthcare Limited, Amersham Hyperfilm ECC).

### Bioinformatics

The 3D X-ray structure of the *T. roseopersicina* Hyn hydrogenase: is not available although previous modeling indicated a high degree of homology between the Hyn hydrogenase of *Thiocapsa roseopersicina* and that of the *Desulfovibrio* enzymes [31]. In this study the high resolution (1.50 Å) X-ray structure of the reduced form of [NiFe] hydrogenase of *Desulfovibrio vulgaris* Miyazaki F (PDB ID: 1WUL [32]) has been chosen as a model for the large subunit structural analysis, since the sequence identity between the two hydrogenases is considerable (sequences of *T. roseopersicina* HynL and *D. vulgaris* HynB are 45.5% identical, 17.6% strongly similar and 11.6% weakly similar.)

This 1WUL model also includes the coordinates of oxygen atoms of the structural water molecules. The entire protein structure has been complemented with the missing hydrogen positions in order to characterize the proper protonation microstates of the protein at pH 7.4. It was also necessary to determine the preferred orientation of the structural water molecules. All of these have been done by the Protonate3D module of the MOE program [46] which is able to predict automatically the hydrogen coordinates at given positions of the heavy atoms of a macromolecular structure. The rotamer, tautomer and ionization state calculation can be put into a single context based upon discrete states. The procedure of Protonate3D solves this discrete formulation of the protonation problem by selecting a protonation state for each chemical group that minimizes the total free energy of the system. During estimation of the protonation microstates, besides flipping terminal amide groups, the position of all the heavy atoms is constrained and only hydrogen atoms were inserted in the appropriate positions. In the both [4Fe4S] clusters, ionization state of the 2-2 iron atoms was kept fixed with the value of +2 and +3, respectively. In the [3Fe4S] cluster, two irons were treated as +3 and remaining one was fixed at the ionization states of +2 [23]. All the cysteine linked to the iron-sulfur clusters or the NiFe active site was fixed in ionization state of −1. Other sulfur atoms in the iron-sulfur clusters were considered as such that they are in the ionization state of −2, while value of +2 was assigned to both Ni and Fe in the active site [47]. The partial charges were assigned according to those of the MMFF94 force field [48]. There is no change in the position of heavy atoms during Protonate3D procedure, since it only introduces hydrogen atoms.

Previously, it has been shown that Protonate3D can accurately predict the location of hydrogen atoms in macromolecular structures [49]. Next, we focused on the strongest non-covalent bonds, the hydrogen bonds and their network. Hydrogen bonds were identified by using the following structural criteria: donor and acceptor atoms must be within 3.5 Å, and the angle between the vector connecting the donor and acceptor atoms and the vector connecting the hydrogen and the acceptor atom must be less than 30 degrees [50]. The hydrogen bond analysis was performed with the VMD program [51]. Numbering the residues and water molecules was done in the same way as in the case of 1WUL.pdb as well as the 3-letter codes of amino acids were adopted from the AMBER nomenclature. It means, that histidine is assigned due to its protonation microstate, namely histidine with hydrogen on the delta nitrogen (HID), with hydrogen on the epsilon nitrogen (HIE) and with hydrogens on both nitrogens (HIP). The imidazole ring of HIP bears two NH bonds, therefore it is positively charged, while HID and HIE are neutral.

## Supporting Information

Figure S1 Superposition of the conserved histidines using hydrogenase structures of *Desulfovibrio vulgaris* Miyazaki F (1WUL.pdb) and the homology model of *Thiocapsa roseopersicina* (HynL.pdb). Amino acids in red represent the conserved histidine residues of *Desulfovibrio vulgaris* Miyazaki F HynB. The green color stands for histidine residues of *Thiocapsa roseopersicina* HynL. The surrounding protein matrix is shown in gray.
(TIF)

Figure S2 Superposition of the proposed proton transfer pathway based on the structure of *Desulfovibrio vulgaris* Miyazaki F. (1WUL.pdb) and the homology model of *Thiocapsa roseopersicina* (HynL.pdb). Amino acids in red represent the conserved histidine residues of *Desulfovibrio vulgaris* Miyazaki F HynB. The green color stands for histidine residues of *Thiocapsa roseopersicina* HynL. The surrounding protein matrix is shown in gray.
(TIF)
