# Supplementary figures and images for: Analyses of the Large Subunit Histidine-Rich Motif Expose an Alternative Proton Transfer Pathway in [NiFe] Hydrogenases
Source: PLoS One. 2012 Apr 12;7(4):e34666. doi: 10.1371/journal.pone.0034666 (PMC3325256; doi:10.1371/journal.pone.0034666)

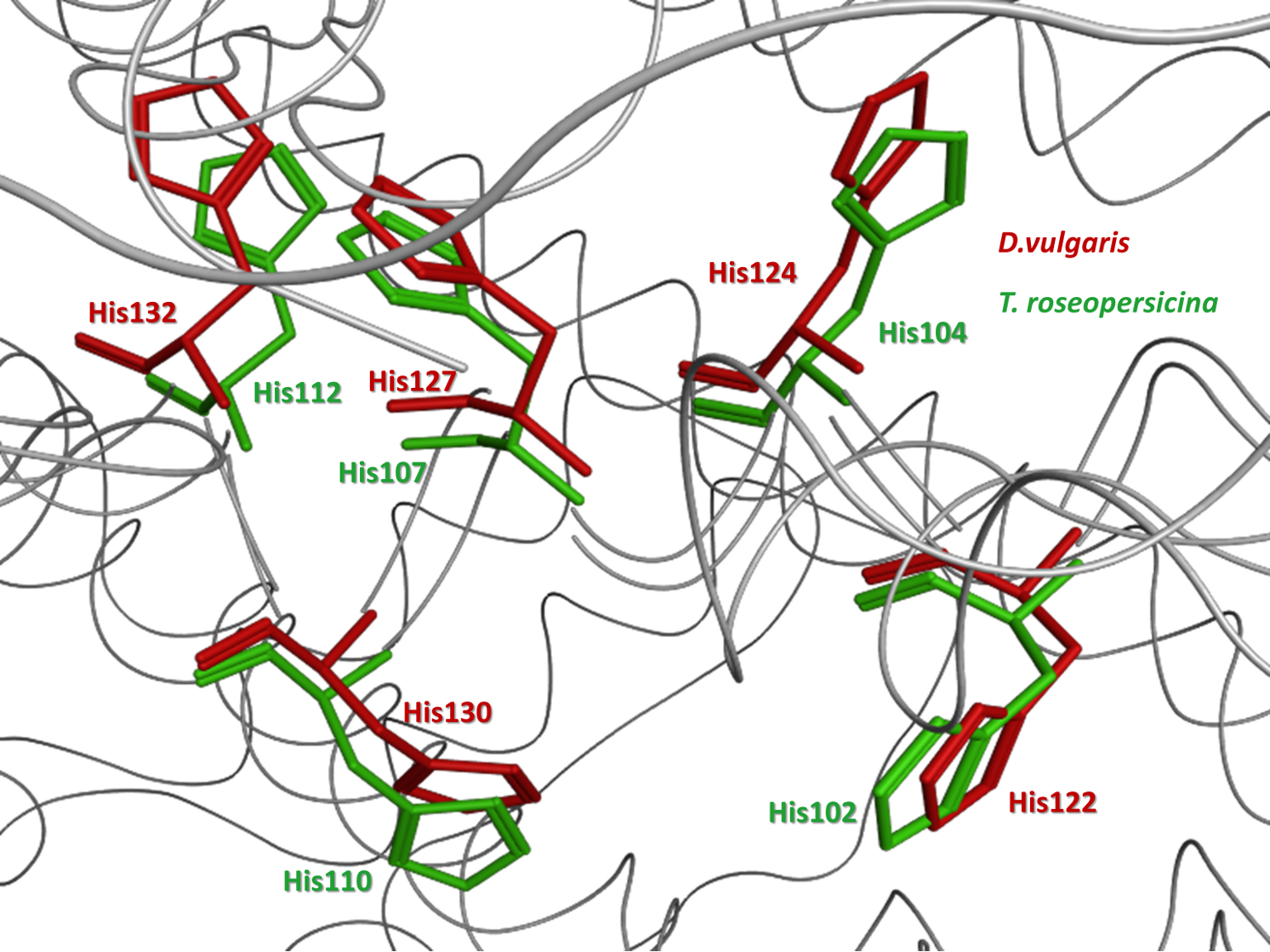

Supplement: Figure S1 — Superposition of the conserved histidines using hydrogenase structures of Desulfovibrio vulgaris Miyazaki F (1WUL.pdb) and the homology model of Thiocapsa roseopersicina (HynL.pdb). Amino acids in red represent the conserved histidine residues of Desulfovibrio vulgaris Miyazaki F HynB. The green color stands for histidine residues of Thiocapsa roseopersicina HynL. The surrounding protein matrix is shown in gray. (TIF) [file pone.0034666.s001.tif]

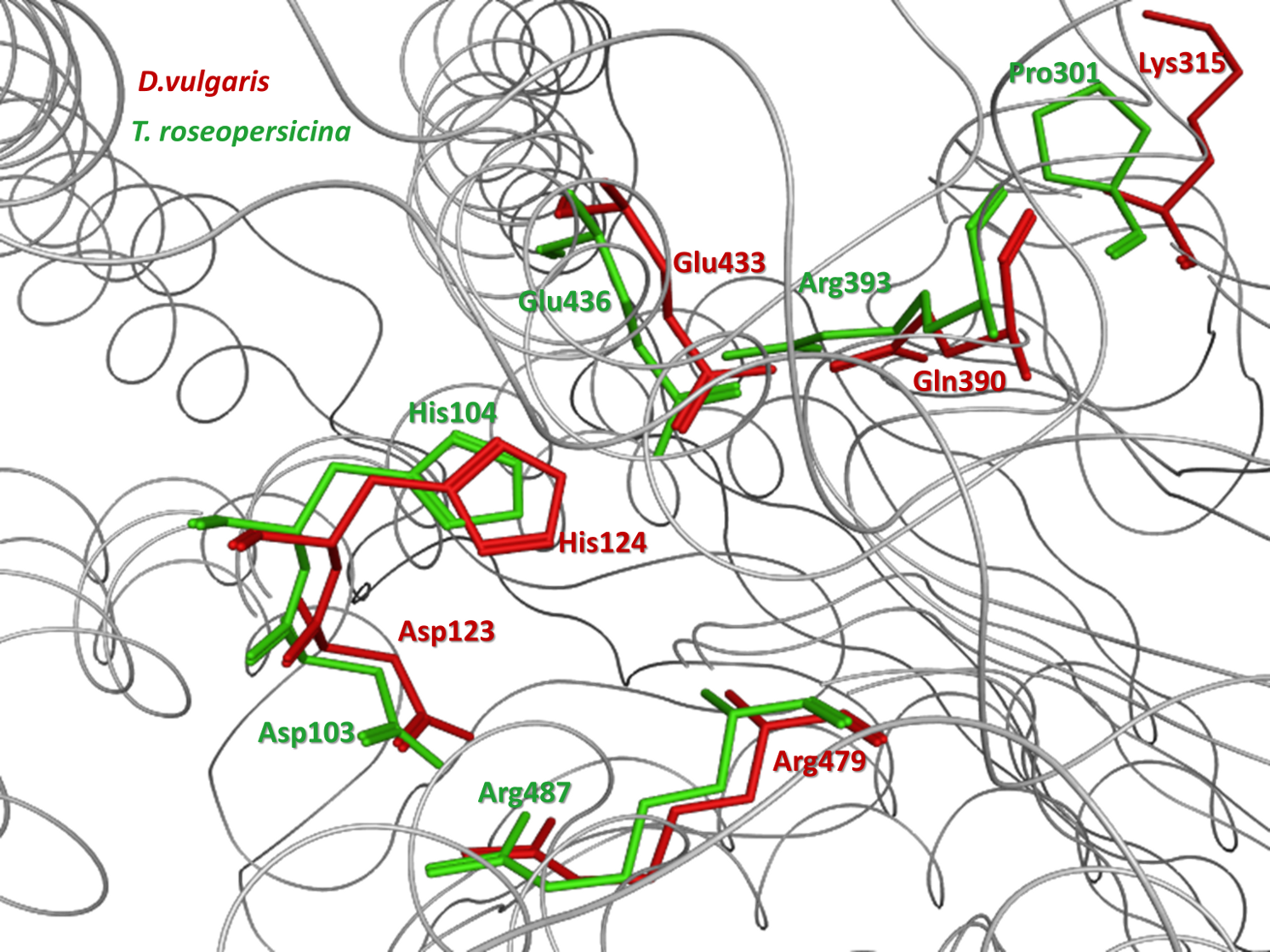

Supplement: Figure S2 — Superposition of the proposed proton transfer pathway based on the structure of Desulfovibrio vulgaris Miyazaki F. (1WUL.pdb) and the homology model of Thiocapsa roseopersicina (HynL.pdb). Amino acids in red represent the conserved histidine residues of Desulfovibrio vulgaris Miyazaki F HynB. The green color stands for histidine residues of Thiocapsa roseopersicina HynL. The surrounding protein matrix is shown in gray. (TIF) [file pone.0034666.s002.tif]
